# Sport participation and gambling-related problems in adolescents: findings from a large school-based study

**DOI:** 10.3389/fpubh.2026.1931143

**Published:** 2026-10-14

**Authors:** Dragana Ratković, Vladimir Knežević, Aleksandra Dickov, Svetlana Ivanović-Kovačević, Predrag Savić, Olga Ivetić, Sanja Bjelan, Maša Čomić

**Affiliations:** University of Novi Sad, Faculty of Medicine Novi Sad, Department of Psychiatry and Psychological Medicine, Novi Sad, Republic of Serbia

**Keywords:** adolescent gambling, behavioral addiction, gambling-related problems, public health, school-based study, sport participation, sports betting

## Abstract

**Background:**

Adolescent gambling is increasingly shaped by digital access, sports betting, and gambling-like mechanisms embedded in everyday youth culture. This study examined gambling-related problems among high school students and their associations with sociodemographic, family, mental health-related, and behavioral characteristics, with particular attention to sport participation.

**Methods:**

A school-based cross-sectional study was conducted among 2,379 high school students in Novi Sad, Serbia, between 2024 and 2026. Participants completed a structured sociodemographic and behavioral questionnaire and the Serbian version of the Gambling Disorder Identification Test. Students were classified as non-gambling, recreational gambling, or having gambling-related problems. Associations between gambling categories and selected characteristics were examined using omnibus chi-square tests, Cramér's V, and *post-hoc* pairwise comparisons.

**Results:**

Overall, 1,969 students (82.8%) were classified as non-gambling, 314 (13.2%) as recreational gambling, and 96 (4.0%) as having gambling-related problems. Gambling category was significantly associated with male sex, lower school achievement, living arrangement, parental marital status, sport participation, previous psychiatric treatment, family psychiatric history, smoking, alcohol use, and psychoactive substance use. In the univariate analyses, sport participation increased from 64.0% among non-gambling students to 75.2% among recreational gamblers and 78.1% among students with gambling-related problems. *Post-hoc* comparisons showed that sport participation differentiated non-gambling students from both gambling-involved groups, but not recreational gambling from gambling-related problems. However, after adjustment for sex and other relevant behavioral and mental health-related characteristics, sport participation was no longer independently associated with gambling-related problems. Male sex, previous psychiatric treatment, smoking, alcohol use, and psychoactive substance use remained the strongest independent correlates.

**Conclusion:**

Gambling-related problems in adolescents were associated with a broader pattern of behavioral and mental health-related factors. Although sport participation was associated with gambling involvement in unadjusted analyses, this association did not remain significant after adjustment for sex and other relevant characteristics. These findings support understanding adolescent gambling within a broader risk-behavior and public health framework, with sport representing a relevant context for prevention rather than an independent risk factor.

## Introduction

Gambling has increasingly shifted from a venue-based adult activity to a highly accessible digital behavior embedded in everyday entertainment, sport, gaming, and social media. Contemporary gambling products are available through mobile phones, operate continuously, and often include rapid betting cycles, live betting, immediate feedback, personalized offers, and repeated engagement. These changes have contributed to the growing view that gambling should be approached as a public health issue, not only as an individual behavioral problem or a narrowly defined clinical disorder (1–3). Although gambling disorder is classified among addictive disorders in current diagnostic systems, gambling-related harm extends beyond individuals who meet full diagnostic criteria (4, 5). Early initiation, repeated exposure, recreational gambling, and subclinical symptoms may all represent points on a wider continuum of risk.

Adolescents are particularly important within this continuum. Although legal gambling is restricted to adults in many countries, adolescent gambling remains common, with estimates varying according to age, gambling type, time frame, and measurement instrument (2, 6, 7). The contemporary gambling environment also weakens traditional boundaries between gambling, gaming, sport spectatorship, and social media. Young people may encounter gambling not only through betting shops, casinos, or lotteries, but also through smartphones, online games, sports content, influencers, and peer communication. This is especially relevant because adolescence is a developmental period marked by increased reward sensitivity, novelty seeking, social evaluation, and peer influence, while inhibitory control and long-term consequence evaluation are still maturing (8, 9). Gambling products are structured around uncertainty, intermittent reinforcement, near-miss outcomes, and the possibility of rapid gain, all of which may be particularly engaging during adolescence (10).

Digitalization has also widened adolescents' exposure to gambling-like mechanisms. Loot boxes, gacha systems, skin betting, esports betting, and other randomized digital rewards share structural characteristics with gambling, including payment, chance-based outcomes, variable reinforcement, and repeated opportunities to spend (11–15). Although not all of these activities meet legal definitions of gambling, they may normalize chance-based spending and increase familiarity with gambling-like reward structures. This matters because adolescent gambling risk is no longer limited to traditional gambling products. It is increasingly shaped by a broader commercial and digital ecosystem in which gambling, gaming, and sport overlap.

Sport occupies a complex position in adolescent development. On one hand, sport participation is usually viewed as protective. It can support physical health, structured leisure time, discipline, self-esteem, social belonging, and prosocial peer affiliation (16). School and community sport may also provide adult supervision and reduce unstructured time, which are traditionally considered protective against several adolescent risk behaviors. On the other hand, the contemporary sport environment is closely intertwined with betting culture. Sports betting is often presented as a natural extension of following sport, discussing sport, or demonstrating sport knowledge. Betting brands are visible through sponsorship, match-day advertising, odds integration, media coverage, digital promotions, and social media content (17–19). As a result, adolescents may encounter gambling not as a separate adult activity, but as part of the wider culture of sport spectatorship, fandom, and sport identity.

At the neurobiological level, sport and gambling both involve reward, motivation, and reinforcement systems. Physical exercise has been associated with structural and functional brain changes and with modulation of neurobiological processes involved in cognition and wellbeing (20). This may be relevant in adolescence, when reward-seeking behavior and striatal reward circuitry have heightened developmental salience (9). This does not imply that sport is harmful in itself. Rather, sport-related reward, competition, and excitement may create a context in which betting is experienced as especially salient.

This creates the central paradox of the present study: sport may be protective in many domains, while also placing adolescents closer to a social and commercial environment in which gambling is normalized. For adolescents who participate in sport, betting may be framed through competition, prediction, team loyalty, and perceived expertise. The belief that one “understands the game” may increase perceived control and reduce the perception that betting is primarily chance-based. In sports betting, knowledge of teams, players, form, and statistics can create an illusion of skill, even when outcomes remain uncertain and odds are commercially structured (10, 18, 19, 21). In this sense, sport participation should not be interpreted as harmful in itself. Rather, sport may represent a relevant context for examining adolescents' exposure to betting visibility, digital access, peer norms, and other risk behaviors.

Evidence linking sport participation and gambling has grown in recent years, although findings differ across sex, sport type, competitive level, and gambling outcome. A longitudinal study of young people found that online gambling increased from late adolescence to early adulthood and that participation in team sports was associated with regular gambling and online gambling among males, independently of sociodemographic and behavioral risk factors (22). Studies among athletes and sports participants have also reported elevated gambling involvement in some competitive sport contexts, particularly where betting is socially visible or embedded in team and spectator culture (23, 24). These findings suggest that sport participation may capture more than individual traits such as competitiveness or sensation seeking. It may also reflect exposure to environments in which sports betting is familiar, socially acceptable, and perceived as part of sport culture.

Adolescent gambling also rarely occurs in isolation. It often appears within a wider cluster of risk behaviors, including alcohol use, tobacco use, psychoactive substance use, school difficulties, and mental health vulnerabilities (25, 26). These associations may reflect shared mechanisms such as impulsivity, sensation seeking, stress coping, peer affiliation, or impaired self-regulation. They may also reinforce one another: alcohol or substance use can lower inhibition and increase risk-taking during gambling, while gambling-related stress, losses, or secrecy may contribute to further substance use or emotional distress. For this reason, sport participation should not be examined as a standalone characteristic. Its association with gambling-related problems needs to be considered alongside sociodemographic, family, school, mental health-related, and substance-use variables.

Serbia represents a particularly relevant setting for examining adolescent gambling because sports betting is well established and highly accessible within the national gambling environment. A nationally representative survey of 5,385 Serbian adults found that sports betting was among the gambling activities most strongly associated with problem gambling, together with casino games and slot machines (27). A subsequent analysis of the same national dataset showed that 10% of adults had participated in sports betting during the previous 30 days. Sports betting was strongly associated with male sex, younger age, urban residence, binge drinking, and an increased likelihood of low/moderate-risk and problem gambling (28). The authors also described the substantial growth and widespread urban availability of sports betting venues in Serbia, suggesting that betting has become a visible and normalized component of the local leisure environment (28).

Although participation in games of chance is legally prohibited for persons younger than 18 years in Serbia, adolescents may nevertheless be exposed to gambling-related messages in their everyday environment. Serbian legislation permits the marketing and advertising of licensed games of chance, while requiring advertisements to include warnings concerning the prohibition of participation by minors and the prevention of gambling addiction (29). Empirical evidence specifically concerning Serbian adolescents remains limited. The Serbian sample of the 2011 European School Survey Project on Alcohol and Other Drugs included 6,084 16-year-old students, of whom 3.1% met the threshold for probable problem gambling on the Lie/Bet screening instrument (30). Although differences in instruments, time frames, and classification criteria preclude direct comparison with the present findings, these data indicate that gambling-related problems among Serbian adolescents had already been identified more than a decade ago. However, despite these important contributions, evidence from Serbia remains limited. Previous studies have primarily focused on gambling prevalence or general sociodemographic correlates, whereas considerably less attention has been given to sport participation or to the simultaneous contribution of family, school, mental health-related, and substance-use factors.

Despite growing international interest, several gaps remain. School-based adolescent samples are still less represented than adult, university, or elite athlete samples. Many studies examine gambling participation rather than severity categories or gambling-related symptoms. Evidence from South-Eastern Europe remains limited, despite the high visibility of betting shops, online gambling, and sports betting advertising in the region. In addition, few studies examine sport participation together with family, school, mental health-related, and substance-use factors in the same adolescent sample.

The present study addresses these gaps by examining gambling-related categories among high school students and their associations with sociodemographic, school, family, mental health-related, and behavioral characteristics. Sport participation is considered as one of these characteristics, within a contemporary environment where sport, betting, digital media, and adolescent peer culture increasingly overlap. By identifying characteristics associated with gambling-related problems in a large school-based adolescent sample, this study aims to inform public health approaches to early identification, school-based prevention, and targeted interventions in youth and sport settings.

## Materials and methods

### Study design and setting

This school-based cross-sectional public health study was conducted among high school students in Novi Sad, Serbia, between 2024 and 2026. Data were collected in four public high schools: one general secondary school (grammar school) and three vocational secondary schools (the School of Economics, the School of Electrical Engineering, and the Secondary Medical School). At the time of the study, Novi Sad had 16 public secondary schools (four grammar schools and 12 vocational schools), serving approximately 18,000 students. The four participating schools therefore represented 25% of all public secondary schools in the city, while the final analytical sample comprised approximately 13% of the local secondary-school student population (31). Both grammar and vocational schools were intentionally included to obtain a heterogeneous sample of students from different educational profiles and academic orientations.

Students from all four grades of secondary education were eligible to participate. Grade-level distribution was not collected during data collection and was therefore unavailable for analysis. Participants were grouped according to chronological age because chronological age was selected as the primary analytical variable, whereas students within the same school grade may differ in age due to differences in school entry or grade repetition. The study examined gambling-related categories and their associations with sociodemographic, school, family, mental health-related, and behavioral characteristics relevant to adolescent risk behavior and prevention.

### Participants

The target population consisted of students attending the participating high schools during the study period. All students who were present in the selected classes on the day of survey administration were invited to participate. The participating classes were selected in collaboration with school administrations according to organizational feasibility and class availability during the data collection period. No formal sampling quotas or probabilistic sampling procedures were applied. Participation was voluntary, anonymous, and without any incentives.

Students were eligible if they were able to complete the questionnaires independently. Students who chose not to participate were not required to provide a reason. Questionnaires with incomplete responses that prevented classification into the predefined GDIT categories were excluded from the final analyses. Completed questionnaires were also reviewed for response quality and internal consistency of response patterns. Questionnaires showing clearly invalid or implausible response patterns, including systematic straight-lining, were excluded from the analyses. The number of questionnaires excluded specifically on the basis of these quality checks was not recorded separately. The final analytical sample comprised 2,379 students.

Information on the total number of students invited to participate, the number of refusals, blank questionnaires, partially completed questionnaires, invalid questionnaires, and other response-rate indicators was not prospectively recorded during data collection and is therefore unavailable. Consequently, formal response-rate calculations and assessment of potential non-response bias were not possible.

### Data collection procedure

Before data collection, approval to conduct the study was obtained from the principals of all participating schools. Survey administration was organized in collaboration with school staff and conducted during regular school hours.

Questionnaires were administered in paper-and-pencil format in classroom settings. Before completion, students received standardized verbal and written information regarding the purpose of the study, the voluntary nature of participation, anonymity, and confidentiality. They were informed that participation was entirely voluntary and that they could decline participation or discontinue questionnaire completion at any time without consequences.

An investigator was present throughout each survey session to provide clarification if necessary, without influencing participants' responses. Completed questionnaires contained no directly identifying information and were analyzed only in aggregated form.

### Measures

#### Sociodemographic and behavioral questionnaire

A structured sociodemographic and behavioral questionnaire was developed for the purposes of this study. It collected demographic, school-related, family-related, mental health-related and behavioral variables relevant to adolescent gambling and associated risk patterns.

The questionnaire included sex, age, place of residence, school achievement, living arrangement, parental marital status, sport participation, previous psychological help, previous psychiatric treatment, family history of psychiatric treatment, smoking, alcohol use, and psychoactive substance use. Most items were dichotomous or multiple-choice, with additional open fields where clarification was needed, such as type and frequency of psychoactive substance use or previous psychiatric treatment.

#### Gambling disorder identification test

The Gambling Disorder Identification Test (GDIT) was used to assess gambling behavior and gambling-related symptoms. Participants completed the official Serbian-language version of the GDIT, which is publicly available through the international GDIT initiative for research and clinical use (32, 33). The Serbian version was developed within the international GDIT translation initiative and was used without modification in the present study.

The GDIT is a 14-item self-report instrument consisting of three domains: gambling behavior (Items 1–3), gambling symptoms (Items 4–10), and gambling-related negative consequences (Items 11–14). It assesses gambling frequency, time spent gambling and thinking about gambling, impaired control, chasing losses, gambling beyond intended limits, concealment of gambling, borrowing money or selling possessions to gamble, gambling to escape negative emotions, tolerance, and financial, interpersonal, mental health, and school/work consequences. For example, one item asks: “During the past 12 months, how often have you tried to control or stop gambling?”

Items 1–3 are scored from 0 to 6, Items 4–10 from 0 to 4, and Items 11–14 from 0, 2, or 4, yielding a maximum total score of 62. Gambling expenditures and types of gambling are assessed separately in an appendix to the instrument and are not included in the total score.

The GDIT was scored according to the original scoring recommendations. Participants with a total score of 0 were classified as non-gambling, those scoring < 15 as recreational gambling, those scoring 15–19 as problem gambling, and those scoring ≥20 as gambling disorder. For the purposes of the present analyses, participants classified as having problem gambling and gambling disorder were combined into a single gambling-related problems group because both categories represent clinically relevant gambling-related harm and the number of participants in the higher-severity categories was insufficient for reliable separate analyses.

The original psychometric evaluation demonstrated excellent internal consistency, good test–retest reliability, and strong discriminative validity for identifying different levels of gambling severity (32). Internal consistency of the GDIT was assessed in the present sample using Cronbach's alpha. The scale demonstrated excellent internal consistency (Cronbach's α = 0.915).

#### Assessment of study variables

Participants completed a structured sociodemographic questionnaire developed for the purposes of this study. The questionnaire collected information on sex, age, place of residence (urban/rural), school type, academic achievement, living arrangement, and parental marital status. In addition, participants were asked whether they participated in sports (yes/no), whether they had ever sought psychological help (yes/no), whether they had ever received psychiatric treatment (yes/no), and whether any family member had ever received psychiatric treatment (yes/no).

Health-related behaviors were assessed using self-report questions on cigarette smoking (yes/no), alcohol consumption (yes/no), and lifetime use of psychoactive substances (yes/no). Participants who reported alcohol consumption or psychoactive substance use were invited to provide additional information regarding consumption patterns or the type of substance used. These supplementary responses were collected as part of the questionnaire but were not included in the present analyses.

### Variables

The primary outcome was GDIT-based gambling category: non-gambling, recreational gambling and gambling-related problems.

Independent variables included sex, age, place of residence, school achievement, living arrangement, parental marital status, sport participation, previous psychological help, previous psychiatric treatment, family history of psychiatric treatment, smoking, alcohol use and psychoactive substance use.

For analysis, selected variables were grouped to ensure clinically meaningful categories and adequate cell counts. School achievement was grouped as excellent vs. very good or lower. Living arrangement was grouped as living with parents vs. other living arrangements. Parental marital status was grouped as married/cohabiting vs. divorced/widowed. Sport participation, smoking, alcohol use and psychoactive substance use were analyzed as dichotomous variables.

### Statistical analysis

Data were analyzed using IBM SPSS Statistics, version 29.0 (IBM Corp., Armonk, NY, USA). Descriptive statistics were used to summarize sample characteristics and GDIT-based gambling categories. Categorical variables were presented as frequencies and percentages.

Associations between gambling categories and sociodemographic, school, family, mental health-related and behavioral variables were first examined using omnibus chi-square tests in 3 × 2 contingency tables. The three gambling categories were non-gambling, recreational gambling and gambling-related problems. For each variable, the omnibus test was used to determine whether the distribution differed across gambling categories.

Because of the large sample size, statistical significance was interpreted together with effect size. Cramér's *V* was calculated for each omnibus chi-square test to estimate the strength of association. Values below 0.10 were interpreted as negligible, values around 0.10 as weak, values around 0.30 as moderate and values around 0.50 as strong associations.

For variables identified as relevant in the omnibus analyses, *post-hoc* pairwise comparisons were performed to determine where the differences occurred. Pairwise comparisons were conducted between non-gambling and recreational gambling, non-gambling and gambling-related problems, and recreational gambling and gambling-related problems.

To assess whether the observed association between sport participation and gambling-related problems was independent of sex and other potential confounders, an additional multivariable logistic regression analysis was performed. For this analysis, the outcome was dichotomized as gambling-related problems (1) vs. no gambling-related problems (0), with the latter category comprising both non-gambling and recreational gambling participants. Variables entered into the model included sex, sport participation, smoking, alcohol use, psychoactive substance use, previous psychological help, previous psychiatric treatment, and family psychiatric history.

All tests were two-tailed. Statistical significance was set at *p* < 0.05.

### Ethical considerations

The study was approved by the Ethics Committee of the Faculty of Medicine, University of Novi Sad, Serbia. The study was conducted in accordance with the Declaration of Helsinki and applicable national regulations.

Written approval was obtained from the directors of all participating schools before data collection. Because the study involved adolescents and sensitive questions related to gambling, mental health, and substance use, data were collected anonymously and reported only at group level. Students were informed that they could refuse participation or stop completing the questionnaire at any time without consequences. No directly identifying personal data were collected. Consent procedures were conducted in accordance with the ethics approval and applicable national regulations.

## Results

### Sample characteristics

The final analytical sample included 2,379 high school students. The sample included 1,179 male students (49.6%) and 1,200 female students (50.4%). The mean age was 16.85 years (SD = 1.71). Most participants lived in an urban environment (72.2%), lived with their parents (94.0%), and reported that their parents were married or in a marital/cohabiting relationship (80.7%). Overall, 66.3% of students reported sport participation. Smoking was reported by 30.1%, alcohol use by 50.7%, and psychoactive substance use by 9.1% of students. Sample characteristics are presented in Table 1.

**Table 1:** Sample characteristics and GDIT-based gambling categories.

Variable	Category	n or value	% or SD
Sex	Male	1,179	49.6
	Female	1,200	50.4
Age	Mean ± SD	16.85	1.71
Age group	14–16 years	835	35.1
	17–18 years	1,544	64.9
Place of residence	Urban	1,718	72.2
	Rural	661	27.8
School achievement	Excellent	1,298	54.6
	Very good	867	36.4
	Good	198	8.3
	Sufficient	12	0.5
	Insufficient	4	0.2
Living arrangement	With parents	2,236	94.0
	Tenant	37	1.6
	Student dormitory	78	3.3
	Other	28	1.2
Parental marital status	Married/cohabiting	1,920	80.7
	Divorced/separated	383	16.1
	Widowed	76	3.2
Sport participation	Yes	1,577	66.3
	No	802	33.7
Previous psychological help	Yes	276	11.6
	No	2,103	88.4
Previous psychiatric treatment	Yes	49	2.1
	No	2,330	97.9
Family psychiatric history	Yes	118	5.0
	No	2,261	95.0
Smoking	Yes	716	30.1
	No	1,663	69.9
Alcohol use	Yes	1,207	50.7
	No	1,172	49.3
Psychoactive substance use	Yes	216	9.1
	No	2,163	90.9
GDIT category	Non-gambling	1,969	82.8
	Recreational gambling	314	13.2
	Problem gambling	30	1.3
	Any gambling disorder	66	2.8
Gambling disorder severity	Mild gambling disorder	16	0.7
	Moderate gambling disorder	18	0.8
	Severe gambling disorder	32	1.3

GDIT-based classification showed that 1,969 students (82.8%) were in the non-gambling category, 314 (13.2%) in the recreational gambling category, and 96 (4.0%) in the combined gambling-related problems category. In the original GDIT classification, 30 students (1.3%) were classified in the problem gambling range and 66 (2.8%) in the gambling disorder range. Among those in the gambling disorder range, 16 students (0.7% of the total sample) were classified as mild, 18 (0.8%) as moderate, and 32 (1.3%) as severe.

### Omnibus comparisons across gambling categories

Omnibus 3 × 2 chi-square analyses showed that gambling category was significantly associated with sex, school achievement, living arrangement, parental marital status, sport participation, previous psychiatric treatment, family history of psychiatric treatment, smoking, alcohol use, and psychoactive substance use (Table 2). No significant differences were observed for age group, place of residence, or previous psychological help.

**Table 2:** Omnibus comparisons of selected characteristics across GDIT-based gambling categories.

Characteristic/category shown	Non-gambling *n* (%)	Recreational gambling *n* (%)	Gambling-related problems *n* (%)	chi-square	*p*	Cramer's *V*	Effect size
Male sex	844 (42.9)	251 (79.9)	84 (87.5)	206.49	< 0.001	0.29	Moderate
Age 17-18 years	1,267 (64.3)	214 (68.2)	63 (65.6)	1.74	0.419	0.03	Negligible
Urban residence	1,418 (72.0)	230 (73.2)	70 (72.9)	0.23	0.891	0.01	Negligible
Very good or lower achievement	846 (43.0)	181 (57.6)	54 (56.3)	28.25	< 0.001	0.11	Weak
Other living arrangement	109 (5.5)	21 (6.7)	13 (13.5)	10.68	0.005	0.07	Negligible
Divorced/widowed parents	352 (17.9)	81 (25.8)	26 (27.1)	14.80	< 0.001	0.08	Negligible
Sport participation	1,261 (64.0)	236 (75.2)	75 (78.1)	21.41	< 0.001	0.10	Weak
Previous psychological help	224 (11.4)	37 (11.8)	15 (15.6)	1.62	0.445	0.03	Negligible
Previous psychiatric treatment	28 (1.4)	4 (1.3)	17 (17.7)	121.47	< 0.001	0.23	Moderate
Family psychiatric history	84 (4.3)	14 (4.5)	20 (20.8)	53.49	< 0.001	0.15	Weak
Smoking	506 (25.7)	150 (47.8)	60 (62.5)	112.64	< 0.001	0.22	Moderate
Alcohol use	901 (45.8)	226 (72.0)	80 (83.3)	116.99	< 0.001	0.22	Moderate
Psychoactive substance use	113 (5.7)	65 (20.7)	38 (39.6)	186.19	< 0.001	0.28	Moderate

For each variable, the displayed category is shown for readability. Omnibus tests were calculated using both categories of each dichotomized variable across the three GDIT-based groups.

Based on Cramér's *V*, the strongest associations were observed for sex, previous psychiatric treatment, smoking, alcohol use, and psychoactive substance use. Sport participation was also significantly associated with gambling category, although the strength of association was weak.

### Sport participation across gambling categories

Sport participation was examined separately to further characterize the univariate association observed across gambling categories. Sport participation was reported by 64.0% of non-gambling students, 75.2% of recreational gambling students, and 78.1% of students with gambling-related problems (Table 3).

**Table 3:** *Post-hoc* comparisons of sport participation across GDIT-based gambling categories.

Pairwise comparison	Sport in group 1 *n/N* (%)	Sport in group 2 *n/N* (%)	chi-square	*P*	Cramer's *V*	Effect size
Non-gambling vs. recreational gambling	1,261/1,969 (64.0)	236/314 (75.2)	14.82	< 0.001	0.08	Negligible
Non-gambling vs. gambling-related problems	1,261/1,969 (64.0)	75/96 (78.1)	7.95	0.005	0.06	Negligible
Recreational gambling vs. gambling-related problems	236/314 (75.2)	75/96 (78.1)	0.35	0.554	0.03	Negligible

*Post-hoc* analyses showed significant differences between non-gambling and recreational gambling students and between non-gambling students and students with gambling-related problems. Sport participation did not differ significantly between recreational gambling and gambling-related problems groups.

### *Post-hoc* comparisons for other characteristics

*Post-hoc* pairwise comparisons showed that most differences occurred between non-gambling students and the two gambling-involved groups. Compared with non-gambling students, recreational gambling students had higher proportions of male sex, lower school achievement, divorced/widowed parental status, sport participation, smoking, alcohol use, and psychoactive substance use.

Compared with non-gambling students, students with gambling-related problems had higher proportions of male sex, lower school achievement, living outside the parental home, divorced/widowed parental status, sport participation, previous psychiatric treatment, family history of psychiatric treatment, smoking, alcohol use, and psychoactive substance use.

When recreational gambling and gambling-related problems were compared directly, significant differences were observed for living arrangement, previous psychiatric treatment, family history of psychiatric treatment, smoking, alcohol use, and psychoactive substance use. Sex, school achievement, parental marital status, and sport participation did not significantly differ between these two groups. Full *post-hoc* comparisons are presented in Supplementary Table 1.

The distribution of selected behavioral and mental health-related characteristics across the three GDIT-based gambling categories is shown in Figure 1. The figure illustrates higher proportions of most selected behavioral and mental health-related characteristics among students with gambling involvement, with the most pronounced differences observed for psychoactive substance use, previous psychiatric treatment, and family psychiatric history in the gambling-related problems group.

**Figure 1:**
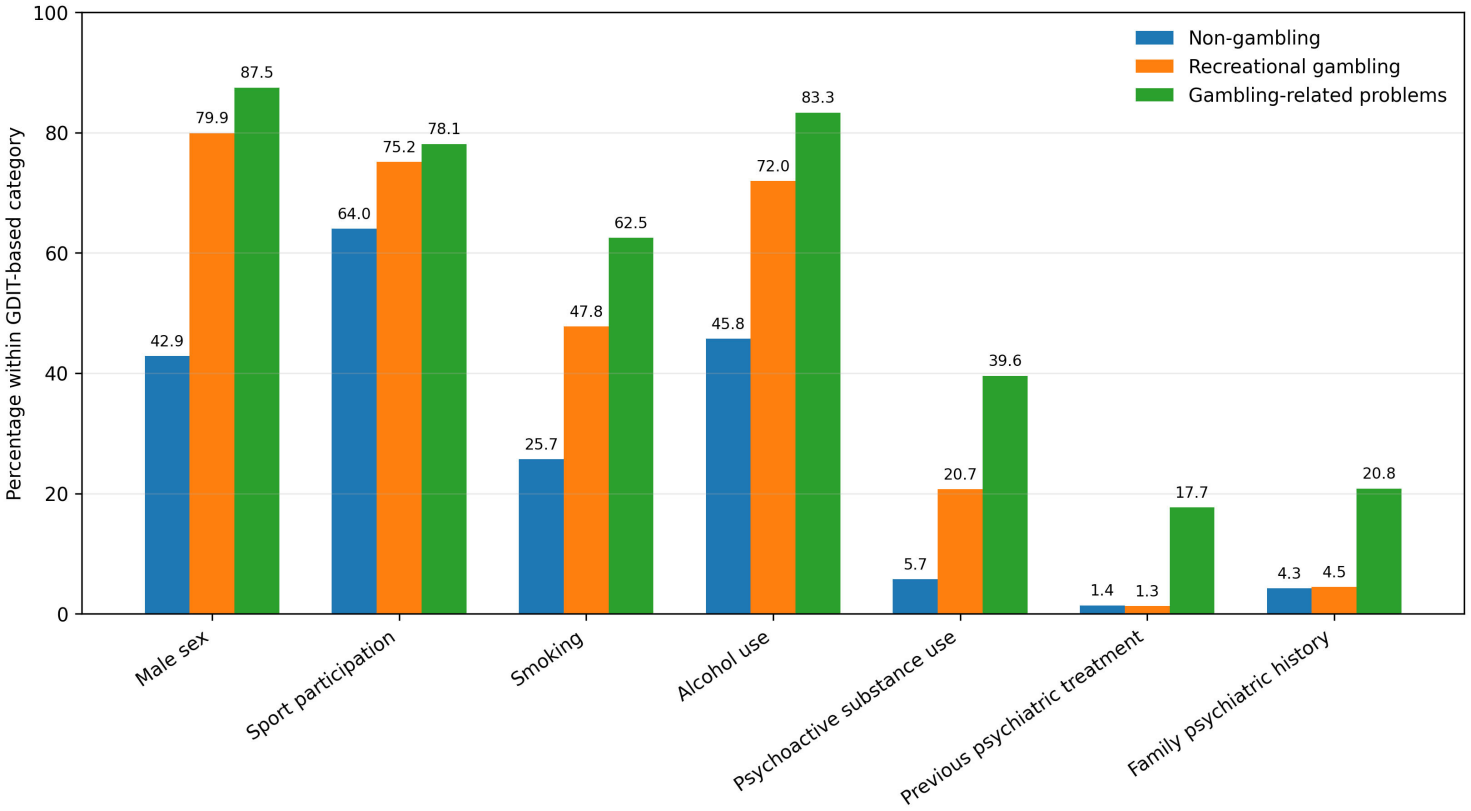
Distribution of selected behavioral and mental health-related characteristics across GDIT-based gambling categories. Values represent percentages within each GDIT-based category.

Because sport participation was significantly associated with gambling-related problems in the univariate analyses, an additional multivariable logistic regression analysis was performed to assess whether this association remained after adjustment for potential confounders. Male sex remained a strong independent predictor of gambling-related problems (adjusted OR 8.85, 95% CI 4.77–16.41; *p* < 0.001), whereas sport participation was no longer independently associated with gambling-related problems after adjustment (adjusted OR 1.33, 95% CI 0.81–2.21; *p* = 0.263). Alcohol use, smoking, psychoactive substance use, previous psychiatric treatment, and family psychiatric history also remained independently associated with gambling-related problems. The results of the multivariable logistic regression analysis are presented in Table 4.

**Table 4:** Multivariable logistic regression analysis of factors independently associated with gambling-related problems.

Variable	Adjusted OR	*p*-value
Male sex	8.85	< 0.001
Sport participation	1.33	0.263
Smoking	2.29	0.001
Alcohol use	2.59	0.002
Psychoactive substance use	2.72	< 0.001
Previous psychological help	0.41	0.053
Previous psychiatric treatment	8.58	< 0.001
Family psychiatric history	3.81	< 0.001

OR, odds ratio. Outcome variable: gambling-related problems (yes/no). Adjusted odds ratios were obtained from the multivariable logistic regression model including all variables listed in the table.

## Discussion

### Principal findings

This school-based cross-sectional study examined GDIT-based gambling categories among 2,379 high school students and their associations with sociodemographic, family, mental health-related, and behavioral characteristics. Although the univariate analyses showed that sport participation was more common among adolescents with gambling involvement, the additional multivariable analysis demonstrated that this association was no longer independent after adjustment for sex and other behavioral and mental health-related characteristics. This represents one of the principal findings of the present study and indicates that the higher prevalence of gambling-related problems observed among adolescents participating in sports is largely explained by male sex and the clustering of correlated risk factors rather than by sport participation itself.

The overall pattern of results supports the view that adolescent gambling should be understood within a broader risk-behavior and public health framework. Recent systematic evidence indicates that gambling and problematic gambling vary considerably across countries and measures, but remain relevant among adolescents and young people (34). European school survey data also show that gambling among adolescents occurs in the same developmental context as alcohol use, smoking, psychoactive substance use, gaming, and other risk behaviors (35). In the present study, gambling category was associated not only with sport participation, but also with male sex, lower school achievement, family-related variables, previous psychiatric treatment, family psychiatric history, smoking, alcohol use, and psychoactive substance use. This suggests that gambling-related problems in adolescence are rarely isolated and should not be approached only as an individual clinical issue.

These findings are consistent with the public health model of gambling, which emphasizes that gambling-related harm is shaped by individual vulnerability, product characteristics, availability, marketing, social norms, and regulatory environments (36). This perspective is especially important for adolescents, who may encounter gambling through sport, gaming, online platforms, social media, and peer communication before they are legally permitted to gamble or fully aware of gambling-related risks.

### Sport participation and gambling-related problems

The univariate analyses showed that sport participation was more frequent among recreational gamblers and students with gambling-related problems than among non-gambling students. However, the multivariable analysis demonstrated that sport participation was no longer independently associated with gambling-related problems after adjustment for sex, smoking, alcohol use, psychoactive substance use, previous psychological help, previous psychiatric treatment, and family psychiatric history. Male sex remained a strong independent predictor. Therefore, the unadjusted association between sport participation and gambling-related problems appears to be largely explained by the overrepresentation of males and the clustering of other behavioral and mental health-related risk factors among adolescents participating in sports.

This finding changes the interpretation of the univariate results. Sport participation should not be considered an independent risk factor for gambling-related problems, nor do the present data indicate that participation in sport directly increases gambling risk. Rather, sport participation may identify a subgroup in which several established gambling-related characteristics are more prevalent. The weak unadjusted association with sport should therefore be interpreted primarily as an expression of correlated demographic and behavioral characteristics rather than as an effect of sport participation itself.

This interpretation does not diminish the established physical, psychological, and social benefits of sport participation during adolescence (16). It does, however, remain relevant that contemporary sport environments are frequently intertwined with sports betting, gambling advertising, sponsorship, televised betting promotions, social media content, and the presentation of betting as an extension of sport knowledge and spectatorship (17–19, 37). Previous research has shown that adolescents' gambling attitudes may be influenced by family, peers, advertising, sport sponsorship, and broader cultural messages that normalize gambling as entertainment (38). Sports betting may be particularly relevant in this context because it directly links gambling with interest in and engagement with sport. Recent research among sports bettors found that sports-specific motives, including knowledge of the game and interest in sports, were associated with more frequent sports betting, highlighting pathways through which engagement with sport may intersect with gambling behavior (39). These contextual exposures may help explain why an unadjusted association with gambling involvement was observed, but they cannot establish that sport participation itself independently contributes to gambling-related problems.

Accordingly, sport settings remain relevant environments for prevention, not because sport participation was identified as an independent risk factor, but because sports clubs, coaches, physical education teachers, and youth sport organizations may provide opportunities to address gambling normalization and identify adolescents with multiple co-occurring risk characteristics. Preventive efforts should focus on gambling literacy, critical understanding of betting advertising and sponsorship, clear messaging regarding underage gambling, and recognition of broader behavioral and mental health-related vulnerabilities rather than on discouraging sport participation.

### Gambling-related problems and broader adolescent risk

The strongest associations in this study were observed for male sex, psychoactive substance use, previous psychiatric treatment, smoking, and alcohol use. This pattern supports the interpretation of adolescent gambling as part of a broader risk-behavior profile. A clear gradient was observed across gambling categories for smoking, alcohol use, and psychoactive substance use, with the highest proportions in the gambling-related problems group.

These findings are consistent with literature showing that gambling and substance use frequently co-occur and may share underlying mechanisms, including impulsivity, sensation seeking, reward sensitivity, stress coping, peer influence, and impaired self-regulation (25, 26, 40). Large-scale data also suggest that gambling involvement is associated with a range of social, financial, and health-related harms, reinforcing the need to consider gambling as part of a wider pattern of adolescent wellbeing and functioning (41). In adolescence, these behaviors may cluster because they are embedded in the same developmental context: peer influence, experimentation, reward seeking, and reduced sensitivity to long-term consequences.

The comparison between recreational gambling and gambling-related problems is particularly informative. In the unadjusted comparisons, sport participation and male sex differentiated non-gambling students from gambling-involved students, but neither characteristic clearly differentiated recreational gambling from gambling-related problems. However, after multivariable adjustment, sport participation was no longer independently associated with gambling-related problems, whereas male sex remained an independent predictor. In contrast, previous psychiatric treatment, family psychiatric history, smoking, alcohol use, and psychoactive substance use were more strongly elevated in the gambling-related problems group. This suggests that the transition from gambling involvement to more harmful gambling may be linked less to sport participation alone and more to cumulative vulnerability, including substance use and mental health-related factors.

Previous psychiatric treatment and family psychiatric history were especially elevated among students with gambling-related problems. This does not mean that gambling-related problems are simply a consequence of psychiatric morbidity. Cross-sectional data cannot determine directionality. However, the finding supports the need to consider gambling within a wider adolescent mental health framework. Behavioral addictions are frequently associated with psychiatric symptoms and substance use, although the direction and mechanisms of these associations remain complex (42). In practical terms, clinicians, school psychologists, and youth mental health services should ask about gambling when adolescents present with substance use, school difficulties, impulsivity, or emotional distress.

### Public health implications

The findings have several implications for prevention. First, adolescent gambling prevention should not focus only on traditional gambling venues. Young people encounter gambling and gambling-like mechanisms through sport, gaming, online platforms, and social media. Prevention should therefore include sports betting, live betting, online gambling, loot boxes, esports betting, gambling advertising, and the ways in which these products are normalized in everyday youth culture.

Second, schools are an appropriate setting for universal prevention. School-based programs can reach adolescents before gambling-related problems become severe or clinically visible. These programs should include gambling literacy, probability and odds education, recognition of cognitive distortions, advertising literacy, and discussion of peer pressure and digital inducements. Evidence from school-based gambling education programs suggests that such interventions can improve gambling-related knowledge and some cognitive outcomes, although stronger evidence is needed regarding long-term behavioral effects (43). Gambling prevention should therefore be integrated into broader health education, together with substance-use prevention, mental health promotion, and digital literacy.

Third, sport settings deserve specific attention. The present findings do not imply that sport should be discouraged. On the contrary, sport should remain part of health promotion. However, sports clubs, coaches, physical education teachers, and youth sport organizations may have a role in preventing the normalization of sports betting among adolescents. Practical measures may include education about betting risks, clear rules against underage betting, critical discussion of gambling advertising in sport, and support for adolescents who show multiple risk markers.

Fourth, prevention should not rely only on individual responsibility. A public health approach to gambling emphasizes that harm is also shaped by commercial and regulatory environments (44). This is particularly relevant when adolescents are exposed to products and marketing strategies designed to increase engagement. Public health regulation may need to address youth exposure to gambling advertising, betting sponsorship in sport, digital promotions, and gambling-like mechanisms in games. Regulatory approaches that separate sport participation from betting promotion may be especially important in adolescent populations (45).

### Strengths and limitations

This study has several strengths, including a large school-based adolescent sample, the use of a structured measure of gambling behavior and symptoms, and the inclusion of sociodemographic, family, mental health-related, and behavioral variables. It also contributes data from South-Eastern Europe, where adolescent gambling remains underrepresented despite the visibility of sports betting and online gambling.

Several limitations should be considered. The cross-sectional design prevents causal inference, and self-reported data may be affected by recall bias, social desirability, or underreporting of sensitive behaviors. Underreporting of gambling may be particularly relevant in this adolescent sample because participation in games of chance is legally prohibited for persons younger than 18 years in Serbia. Sport participation was measured as a dichotomous variable, so the study could not distinguish between team and individual sport, recreational and competitive sport, frequency or intensity of participation, coaching and team environments, or direct exposure to betting within sport settings. Similarly, several mental health-related and behavioral variables were assessed primarily as dichotomous indicators. The analyses therefore did not capture the type, severity, or timing of psychiatric symptoms and treatment, or the frequency, quantity, and timing of smoking, alcohol consumption, and psychoactive substance use. These factors may influence the strength and nature of their associations with gambling-related problems and should be examined in greater detail in future studies. In addition, the GDIT was originally psychometrically evaluated in adult samples; therefore, its categories in this adolescent school-based sample should be interpreted as screening-based indicators rather than formal clinical diagnoses (32).

An additional limitation is that response-rate indicators (including the number of invited students, refusals, and incomplete or invalid questionnaires) were not prospectively recorded during data collection. Consequently, response rate and potential non-response bias could not be assessed.

Although the overall sample was large, only 96 students (4.0% of the total sample) were classified as having gambling-related problems. The relatively small size of this subgroup limits the generalizability of findings concerning gambling-related problems beyond the present study population and school-based sample. These findings should therefore be interpreted with caution and require confirmation in larger and more diverse adolescent samples.

Although an additional multivariable analysis was performed to account for major measured confounders, the cross-sectional design does not allow conclusions regarding temporality or causality. Future longitudinal studies are needed to clarify whether sport participation precedes gambling involvement or whether both reflect shared developmental, social, and behavioral risk factors. Such studies could also better evaluate how changes in sport participation, exposure to gambling marketing, and mental health over time influence the development of gambling-related problems.

This study adds to the evidence that adolescent gambling should be understood within a broader risk-behavior and public health framework. Gambling-related problems were associated with a cluster of behavioral and mental health-related characteristics, while the observed unadjusted association with sport participation did not remain significant after multivariable adjustment. These findings suggest that sport participation should not be interpreted as an independent risk factor, but considered within the broader social and behavioral context in which adolescent gambling occurs.

These findings suggest that adolescent gambling prevention should be integrated into school health promotion, youth mental health, substance-use prevention, and sport settings. Public health strategies should address the normalization of sports betting, improve gambling literacy among adolescents, and involve schools, families, coaches, and policymakers in reducing gambling-related harm before it becomes clinically severe.

## Data Availability

The datasets presented in this article are not readily available because they contain data collected from minors on sensitive behaviors, including gambling, mental health, and substance use, and public sharing could compromise participant anonymity. Requests to access the datasets should be directed to Maša Comić, masa.comic@mf.uns.ac.rs.
